# Tumour of the Orbit

**Published:** 1846-10

**Authors:** 


*Tumour of the Orbit*—Mrs. Rebecca Pope, aged 40 years—of light complexion, spare habit, and the mother of several children.

This lady was brought from Fairfield Co., O , to Columbus, about one year ago, at the time when the Ohio State Medical Convention was in session, for the purpose of consulting some of its members concerning a large and nodulated tumour which completely filled the orbit of the left eye. The history of the case is nearly as follows: At the early age of 12 years, she noticed the sight of the left eye began to fail, and continued to do so until she was 17, when the sight was entirely lost, and the eye itself so prominent that her physicians supposed it to be a dropsical enlargement, for which they punctured the globe. Nothing escaped of consequence, but a violent inflammation supervened, which occasioned a collapse and atrophy of the organ. For about 20 years she experienced no particular inconvenience from it; but about two years previous to her visit to Columbus, she began to feel some uneasiness and pain, together with a decided increase of morbid growth in the socket. The symptoms became so alarming, that she now resolved to seek relief.

At that time she was visited and examined by Drs. Mussey and Judkins, of Cincinnati, and several of the older members of the Convention, who were unanimously of the opinion that the tumour was *probably* malignant, and that the only chance of relief would be found in an immediate extirpation.

The tumour itself completely filled the orbit, and projected considerably beyond the brow and malar prominence. It seemed firmly fixed in its position, and quite immovable. Upon its anterior surface, was imbedded the globe, flattened and diminished to one-eighth its natural size. The palpebræ were widely separated ; and unable to cover the tumour.

In consultation, I agreed with all the other medical gentlemen, that the entire morbid growth should be removed with the knife, and advised her to submit to an operation which would afford a fair, or at least the *only* prospect of cure—to which she with but little delay consented.

With the assistance of my friends Drs. Schenck, Smith, Dennig and Taylor, and in the presence of several medical students, I proceeded, on the 24th of May, 1845, to extirpate the eye and tumour. The patient was placed in an easy chair, with her head thrown back, and held by an assistant. The external commissure of the lids was divided backwards; the bistoury was then passed between the tumour and the walls of the orbit, nearly to the bottom of the socket, and carried carefully around it until it arrived at the place of entrance. A little dissection with the point of the instrument removed the entire contents of the orbit. The hæmorrhage was not profuse, and the patient bore her suffering with remarkable fortitude. The cavity was lightly filled with lint, suitable dressing applied, and the patient laid in bed. As the pain in the part was severe, one-third of a grain of morphine was administered, which soon afforded relief, when she expressed herself as exceedingly gratified that she had submitted to the operation.

*May 25th*, morning.—Rested but little during the night. Nausea and vomiting, pain in the head and general distress had all supervened to an alarming degree. Appropriate remedies were prescribed to allay the irritation of the stomach, and divert the tendency to cerebral disease, but to little purpose. During the afternoon, she exhibited symptoms of approaching delirium, and in the evening her mind was decidedly wandering. Prescribed 20 grs. calomel, in divided doses, sinapisms to the extremities, cold water to the head, and blister to the nape of the neck.

26th.—Calomel had operated freely—mind more calm and collected, but not perfectly rational. A general distress and restlessness pervaded the whole system. No arterial excitement or general reaction had yet taken place. Pulse varied from 90 to 120. During the day, symptoms of cerebral disturbance and organic disease increased. In the evening, at my request, my friends Drs. Parson and Smith saw her with me, in consultation. Similar measures were continued, with the addition of ammonia, and other diffusible stimulants.

27th.—She had no rest during the night; pulse diminished in force and frequency; mind more incoherent and comatose; secretions all scanty and vitiated. Wound examined, and found livid and flabby, and exhibiting no signs of reparation.

These symptoms and others, indicating approaching dissolution, increased, in spite of all treatment, until the 29th, the sixth day after the operation, the patient died, apparently, from inflammation of the brain. To my extreme regret, a post mortem examination could not be obtained.

On examination of the morbid growth, the disease was found to consist of a tumour the size of a small orange, which commenced in the centre of the optic nerve, between its exit from the foramen opticum, and its entrance into the globe of the eye.

As the tumour increased in size, the neuralema and nervous filaments yielded, and became so expanded over the whole periphery of the tumour, as to form a complete tunic. On dividing this covering, the morbid structure was easily separated from it. Its color was nearly that of chocolate, and of the consistence of liver. Its organization was very imperfect, being easily broken down with the fingers and permeated in many parts by irregular sinuosities. Whether or not the idea is correct, it is impossible to determine, but I was impressed with the suggestion that this abnormal growth might have arisen from the organization of a small quantity of blood, which might have been extravasated in the substance of the nerve, occasioned by an early rupture of the *arteria centralis retina.*

This case is interesting in many respects; but particularly that the patient, after a complete and speedy removal of a tumour of the orbit, apparently in no way connected with the brain, should die so suddenly from inflammation of that organ. A past mortem examination would probably have revealed the fact that organic disease has already committed fatal ravages within the cranium.—*Proceedings of the Ohio Medical Convention.*

